# RNAi technology for plant protection and its application in wheat

**DOI:** 10.1007/s42994-021-00036-3

**Published:** 2021-03-11

**Authors:** Shaoshuai Liu, Shuaifeng Geng, Aili Li, Yingbo Mao, Long Mao

**Affiliations:** 1 Institute of Crop Science, Chinese Academy of Agricultural Sciences, Beijing, 100081 China; 2 Chinese Academy of Sciences (CAS) Key Laboratory of Insect Developmental and Evolutionary Biology, CAS Center for Excellence in Molecular Plant Sciences, Shanghai Institute of Plant Physiology and Ecology, University of CAS, Chinese Academy of Sciences, Shanghai, 200032 China; 3 Sino-Agro Research Station for Salt Tolerant Crops, Yellow River Delta, Kenli District, Dongying, 257500 Shandong China

**Keywords:** Double-stranded RNA, Pathogens, Pests, Nematodes, RNA interference, Small RNA, Wheat

## Abstract

The RNAi technology takes advantage of the intrinsic RNA interference (RNAi) mechanism that exists in nearly all eukaryotes in which target mRNAs are degraded or functionally suppressed. Significant progress has been made in recent years where RNAi technology is applied to several crops and economic plants for protection against diseases like fungi, pests, and nematode. RNAi technology is also applied in controlling pathogen damages in wheat, one of the most important crops in the world. In this review, we first give a brief introduction of the RNAi technology and the underneath mechanism. We then review the recent progress of its utilization in crops, particular wheat. Finally, we discuss the existing challenges and prospect future development of this technology in crop protection.

## Introduction

Wheat (*Triticum aestivum* L.) contributes more than 20% of the total dietary calories and proteins for humans worldwide (Shiferaw et al. 2013). It plays a pivotal role in securing the global food demand. The increase of wheat yield, however, has slowed down in recent years partly due to newly emerging varieties of various diseases—pathogens, pests and nematodes (Rosegrant and Cline 2003). On the other hand, the overuse of pesticides for disease control has posed substantial risks to food safety, the environment, and all living organisms (Ali 2014). The transgenic crops expressing insecticidal proteins from *Bacillus thuringiensis* (*Bt*) effectively reduced the insecticide usage and increased crop yields. However, the limited scope of *Bt* crops and the appearance of *Bt*-resistant pests in fields call for new technologies for pest control (Carriere et al. 2015; Jin et al. 2015; Tabashnik et al. 2013). The phenomenon of RNA interference (RNAi) is widely found in eukaryotes (plants, fungi, insects, animals, and nematodes etc.) and has been developed as a promising technology for crop health protection (Zhang et al. 2017). RNAi is a natural process that involves the regulation of gene expression by several manners: effective post-transcriptional gene silencing (PTGS), translational inhibition, RNA destabilization, and/or transcriptional gene silencing (TGS) by directing DNA methylation (Fire et al. 1998; Coleman et al. 2015; Ghildiyal et al. 2008; Huvenne and Smagghe 2010; Jones-Rhoades et al. 2006; Liu et al. 2020; Mao et al. 2007; Sherman et al. 2015). Here, we review recent progress in the development of RNAi-based plant protection technologies, particularly on its application in wheat. We discuss its potential for the control of fungal pathogens, pests and nematodes, as well as current challenges facing RNAi strategy. We also prospect the future improvement in delivery methods for effective applications of this technology in crop protection.

## The mechanism of RNAi technology

**Fig. 1:**
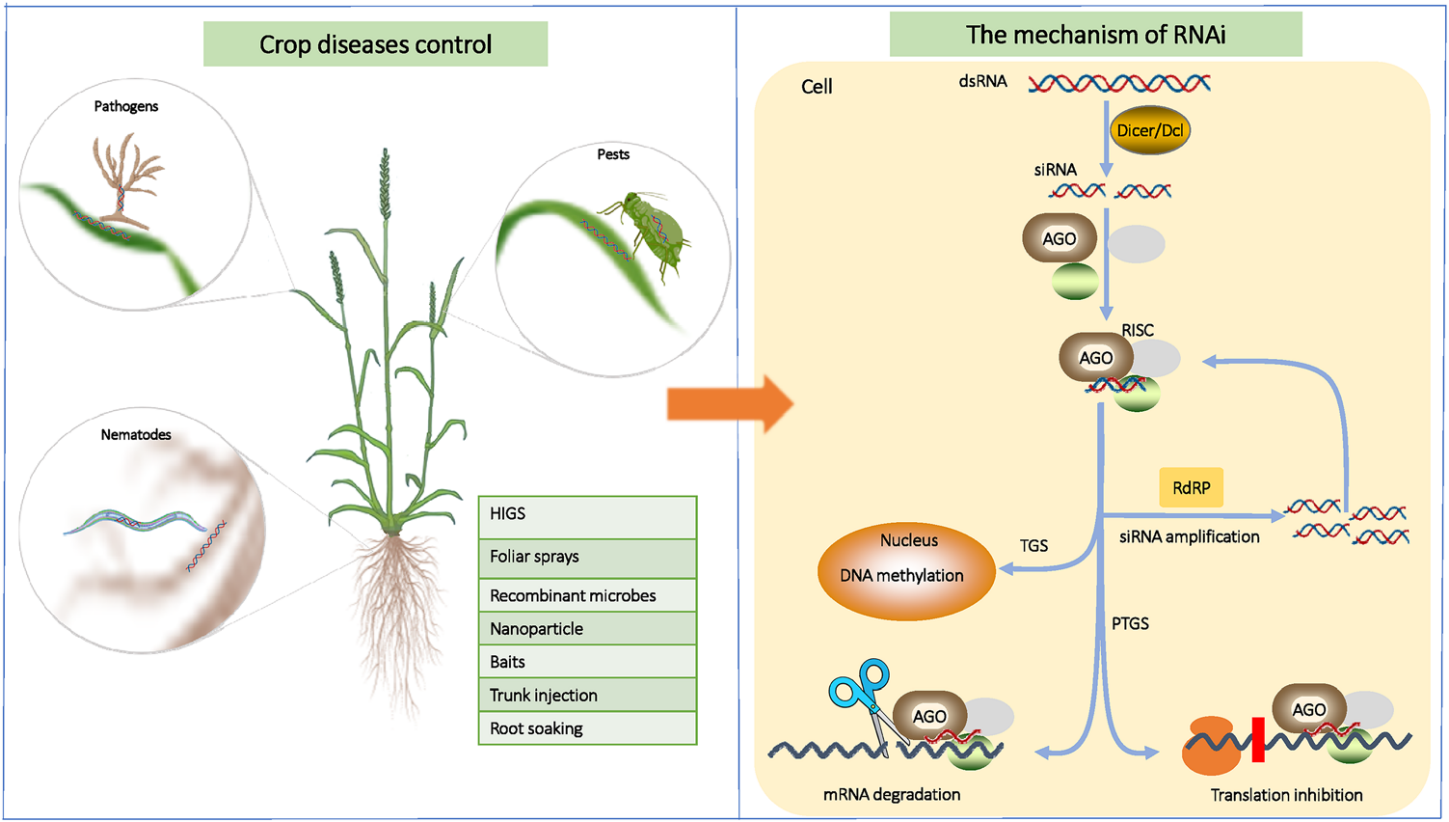
The RNAi technology and its application in crop diseases control. (*Left*) Crop disease control by RNAi. dsRNA delivery strategies for wheat protection mainly via HIGS, foliar sprays and recombinant microbes. Each of these strategies contains advantages, relying on the specific condition involved. Additional methods are also used such as nanoparticles, baits, trunk injection, and root soaking. (*Right*) The RNAi mechanism. Double-stranded RNA (dsRNA) molecule binds to a Dicer/Dcl protein, which cleaves it into small interfering RNAs (siRNAs); these siRNAs bind to an Argonaute (AGO) protein, to form an RNA-Induced Silencing Complex (RISC), then RISC separates the siRNAs into two strands. The siRNA/RISC complex binds the complementary sequence of the target mRNA resulting in post-transcriptional gene silencing (PTGS) via degradation of the mRNA target (indicated by a scissor) or inhibition of its translation (indicated by red vertical bar), or resulting in transcriptional gene silencing (TGS) in the nucleus via chromatin modifications. In fungi and nematodes, the silencing signal can be perpetuated by the action of the RNA-dependent RNA polymerase (RdRP) for siRNA secondary amplification, but for pests, RdRP is not yet found

RNAi is a self-protection mechanism in eukaryotic cells and is triggered by double-stranded RNA (dsRNA) when present in a cell. dsRNA is processed by the ribonuclease III enzyme Dicer or Dicer-like enzymes to produce small interfering RNAs (siRNAs) of 20–30 nucleotide (nt) long. These small RNA (sRNA) are bound to Argonaute family proteins (AGOs), the catalytic components of the RNAi system. The AGO/siRNA complexes are then recruited to the RNA-induced silencing complex (RISC) (Lee et al. 2010), which mediates mRNA degradation, mRNA translation, or chromatin modification (Borges and Martienssen 2015) (Fig. 1). In most eukaryotes, including pathogens and pests, RNA-dependent RNA polymerases (RdRPs) have been identified for secondary dsRNA synthesis and are essential for the systemic effect of RNAi. Two works have specified functions for the RdRP activity in RNAi in *Caenorhabditis elegans* (Sijen et al. 2001) and fungi (Dang et al. 2011); however, a similar RdRP-based amplification system is yet to be discovered in insects (Zotti et al. 2018). Given the presence of RNAi pathways in pathogens, pests, and nematodes, it is not surprising to take advantage of its working mechanism in crop protection.

## Delivery of interfering RNAs

Interspecific transportation of sRNAs takes place naturally. siRNAs can be shuttled between plants and pathogens by secreted vesicles (Cai et al. 2018; Weiberg et al. 2013). In cotton, the production of microRNAs (miRNAs) miR166 and miR159 was increased upon *Verticillium dahliae* (a vascular fungal pathogen responsible for devastating wilt diseases in many crops) infection and transported to infection sites to silence virulence genes reducing its damage (Zhang et al. 2016). Despite these in vivo mechanisms, the RNAi technology is impeded by in vitro dsRNA delivering efficiency. Numerous efforts on artificial delivery methods have been attempted. The selection of the suitable delivery approaches (e.g. host-induced gene silencing, foliar sprays, recombinant microbes) is in fact determining the success of the technology (Fig. 1). A few methods have been tested.

The first approach is the application of synthetic dsRNA or sRNA derived from pathogen or pest genes as pesticides on crop leaves. Foliar application with sprayable RNAi-based products, such as sRNAs, is suitable for controlling pests and pathogens on stems, foliage, or fruits. The products are evaluated similarly to topical pesticides where the RNA solution is sprayed on leaves, or fed to the target pests, and impacts on insects are then observed (Andrade and Hunter 2017). One of the first case exploring the applications of sprayable RNA molecules to control pests was the use of siRNA against the diamondback moth (*Plutella xylostella)*. *Brassica* leaves that were sprayed with chemically synthesized siRNAs targeting the *acetylcholine esterase* gene *AchE2* caused high mortality for *P. xylostella*. (Gong et al. 2013). In another case, foliar application of dsRNA targeting the *cytochrome P450* (*CYP3*) gene of *Fusarium graminearum* resulted in successful inhibition of fungal growth in directly sprayed leaves as well as the distal non-sprayed leaves in barley plants (Koch et al. 2016). This strategy or so-called spray-induced gene silencing (SIGS) opens an avenue of development of biopesticide which is environmentally friendly. Moreover, since RNAi is highly dependent on the sequence specificity, it has little effects on the non-target microorganisms or non-target pests.

The second method is to use recombinant microbes such as virus and bacteria engineered to produced dsRNA in host crops (Cagliari et al. 2018; Dubrovina and Kiselev 2019; Goulin et al. 2019). Virus-induced gene silencing (VIGS) is a naturally occurring (Baulcombe 2015; Waterhouse et al. 2001). Unlike stable RNAi and mutants, the transiently expressed dsRNA by VIGS does not modify plant genetic composition. For instance, three midgut-expressed *CYP* genes of the Lepidoptera insect, *Manduca sexta* were targeted through viral vectors to produce dsRNA in the host plant. The viral vector was engineered using *Tobacco Rattle Virus* (TRV) to deliver dsRNA into *Nicotiana attenuata* (Kumar et al. 2012). DsRNA could also be produced in the bacteria (HT115). When the cotton bollworm (*Helicoverpa armigera*) larvae fed with the artificial diet coated with dsRNA expressing HT115, high mortality was observed after five days. Data showed that inhibition of target gene expression led to significant reductions in body weight, body length, and pupation rate (Ai et al. 2018).

The third approach is host-induced gene silencing (HIGS) which employs transgenic plants to produce dsRNA derived from pathogen or pest genes. RNAi occurs in pests when they ingest sufficient dsRNA or sRNA. Tests have been made for a few pests where persistent effects were obtained for several common species (Baum et al. 2007; Mao and Zeng 2014; Sun et al. 2019; Zhu et al. 2012). The phloem-feeding hemipterans such as aphids with specialized mouthparts (stylets) that penetrate through plant tissues to ingest cell saps. In this case, dsRNA sequences of *shp* gene effectively reduced the growth, the reproduction, and the survival rate of tested aphids. Remarkably, other developmental aberrations were also observed such as winged adults and delayed maturation (Abdellatef et al. 2015). This method is a complementary tool to *Bt*-based insect-resistant plants which is not effective for several hemipterans with specialized stylets. Cotton plants constitutively expressing dsRNA from genes encoding the P450 protein CYP6AE14 and NDPH dehydrogenase protein 2 of cotton bollworm (*Helicoverpa armigera*) significantly improved resistance to this pest, and the *dsNDPH* cotton is almost equivalent to *Bt* cottons in resistance efficiency (Mao et al. 2011; Wu et al. 2016). Similarly, dsRNA homologous to V-type ATPase gene of corn root worm (*Diabrotica virgifera*) in transgenic corn plants rendered significant improvement of insect resistance (Baum et al. 2007).

For woody plants, such as fruit trees, dsRNA can be delivered via insecticidal baits, nanoparticle trunk injection and root soaking. The information of these methods can be found elsewhere for detail (Liu et al. 2020; Zhu and Palli 2019).

## The application of RNAi for wheat protection

### Management of bacterial and fungal pathogens

In wheat, a few serious wheat diseases, such as Fusarium head blight (FHB) caused by necrotrophic fungi of the genus *Fusarium* and leaf rust caused by biotrophic fungi of the genus *Puccinia* (Table 1), have been targeted using RNAi technology. Transgenic wheat plants were engineered to confer three hairpin RNA fragments derived from the *Fusarium graminearum* chitin synthase gene (*Chs3b*), which is responsible for the biosynthesis of chitin. These transgenic plants showed strong resistance to FHB and Fusarium seedling blight (FSB) (Cheng et al. 2015). On the other hand, expressing dsRNA complementary to mRNAs of *Puccinia triticina* MAP-kinase (*PtMAPK1*, 520 bp) or a cyclophilin (*PtCYC1*, 501 bp) showed efficient silencing of the corresponding genes in the fungus and significant reduction of the fungal pathogenicity and growth in transgenic wheat. *P. triticina* is an aggressive fungal pathogen that causes severe leaf rust disease in wheat. *P. triticina* proliferation was significantly reduced together with decreasing fungal target gene transcript abundance and reduced biomass accumulation in RNAi-based resistant plants (Panwar et al. 2018).

**Table 1:** RNAi target genes tested in pests/pathogens/nematodes

Organism	Target genes	Assay/method	Effects	References
Insects				
*Sitobion avenae *	Salivary sheath protein (*SHP*)	HIGS	Mortality/fecundity/transgenetional gene silencing	Abdellatef et al. (2015)
*Rhopalosiphum padi*	Acetylcholinesterase gene *RpAce1*	Injection	Susceptibility/fecundity	Xiao et al. (2015)
*Sitobion avenae*	Catalase gene *CAT*	Feeding	Mortality	Deng and Zhao (2014)
*Sitobion avenae*	Acetylcholinesterase gene *SaAce1*	Injection	Susceptibility/fecundity	Xiao et al. (2015)
*Sitobion avenae*	Cytochrome *c *oxidase subunit VIIc precursor, zinc finger protein, three unknown proteins	Feeding	Mortality/developmental stunting	Zhang et al. (2013)
*Sitobion avenae*	Secreted salivary peptide *DSR32*, salivary protein *DSR33*, serine protease 1 *DSR48*	Feeding	Mortality	Wang et al. (2015)
*Sitobion avenae*	Olfactory coreceptor gene *SaveOrco*	Feeding	Impaired response	Fan et al. (2015)
*Sitobion avenae*	Lipase maturation factor 2‐like gene	HIGS	Mortality/fecundity	Xu et al. (2017)
*Sitobion avenae*	Laccase 1 (*Lac1*)	Feeding	Mortality	Zhang et al. (2018)
*Sitobion avenae*	Zinc finger protein (*SaZFP*)	HIGS	Mortality/transgenetional gene silencing	Sun et al. (2019)
*Sitobion avenae*	Ecdysone receptor (*EcR*) and ultraspiracle protein (*USP*)	Feeding	Mortality/fecundity	Yan et al. (2016)
*Sitobion avenae*	Chitin synthase 1 (*CHS1*)	HIGS	Mortality/fecundity	Zhao et al. (2018)
Pathogens				
*Fusarium graminearum *	Cytochrome P450 lanosterol C-14*α*-demethylase (*CYP51*)	HIGS	Inhibiting fungal mycelium formation	Koch et al. (2013)
*Fusarium graminearum *	Cytochrome P450 lanosterol C-14*α*-demethylase* CYP51*	SIGS	Inhibition of fungal growth	Koch et al. (2016)
*Fusarium graminearum*	Chs3b	HIGS	Restriction of fungal growth through	Cheng et al. (2015)
*Blumeria graminis*	Virulence effector (*Avra10*)	HIGS	Reduced fungal development	Nowara et al. (2010)
*Fusarium asiaticum *	*Myosin 5 *	SIGS	Reduced virulence	Song et al. (2018)
*B. graminis *f. sp*. hordei*	Ribonuclease-like protein	HIGS	Reduced virulence	Pliego et al. (2013)
	Ribonuclease-like protein			
	Virulence effector			
	Glucanase			
	Metalloprotease			
	Virulence effector			
*Fusarium culmorum*	Secreted lipase (*Fgl1*), Mitogen-activated protein (MAP) kinase (*Fmk1*), Beta 1,3-Glucan synthase (*Gls1*)	VIGS and HIGS	Reduced virulence	Chen et al. (2016)
*Puccinia striiformis *f. sp*. tritici*	Calcineurin homologue (*PsCNA1, PsCNB1*)	VIGS	Slower extension of fungal hyphae	Yin et al. (2010)
*Puccinia striiformis *f. sp*. tritici*	Mitogen-activated protein kinase (*MAPK1*), Cyclophilin (*CYC1*), Calcineurin regulatory subunit (*CNB*)	VIGS	Reduced virulence	Panwar et al. (2013)
*Puccinia striiformis *f. sp*. tritici*	Protein kinase A catalytic subunit (*PsCPK1*)	VIGS	Reduced virulence	Qi et al. (2018)
*Blumeria graminis* f. sp. *tritici*	Three virulence effectors (*SvrPm*^*3a1/f1*^)	HIGS	Reduced virulence	Schaefer et al. (2020)
*Puccinia striiformis *f. sp*. tritici*	Glycine-serine-rich effector (*PstGSRE1*)	HIGS	Reduced virulence and increased H_2_O_2_ accumulation	Qi et al. (2019a)
Nematodes				
*Meloidogyne* *incognita*	Heat-shock protein 90, isocitrate lyase, Mi-cpl-1	HIGS	Reduced reproduction	Lilley et al. (2007)
*Pratylenchus spp.*	Troponin C (*pat-10*) Calponin (*unc-87*)	Soaking solution	Reduced reproduction	Tan et al. (2013)

Powdery mildew caused by *Blumeria graminis f. sp. hordei* in barley and *B. graminis f. sp. tritici* in wheat is a serious disease as well. Transgenic barley expressing dsRNA targeting the avirulence gene *Avra10*, which corresponds to the resistance gene *Mla10,* showed reduced fungal gene transcripts and severely affected fungal development (Nowara et al. 2010). Silencing of 1,3-*β*-glucanosyltransferase genes (*BgGTF1* and *BgGTF2*) via VIGS that was built on the barley stripe mosaic virus (BSMV) significantly slowed down the growth of the powdery mildew fungus (Qi et al. 2019b). Mildew resistance locus o (Mlo) encodes a transmembrane protein (Panstruga et al. 2005) that acts as a negative regulator to suppress plant immunity in uninfected tissues. It is also involved in protection against cell death as well as in responses to biotic and abiotic stresses (Piffanelli et al. 2002). Down-regulation of the *TaMlo* gene via VIGS resulted in the broad-spectrum powdery mildew resistance in wheat (Várallyay et al. 2012). Recently, gene-editing technologies were used to achieve similar effects. For instance, simultaneous knockout of the three *TaMlo* homoeologues by TALEN (transcription activator‐like effector nuclease) produced transgenic wheat plants that were highly resistant to powdery mildew infection, another work produced transgenic wheat plants that carry mutations in the *TaMLO-A1* allele using the CRISPR-Cas9 technology (Wang et al. 2014). On the other hand, non‐transgenic TILLING (Targeting Induced Lesions IN Genomes) plants with partial loss‐of‐function alleles of *TaMlo* confer durable broad‐spectrum powdery mildew resistance (Acevedo-Garcia et al. 2017).

Wheat *streak mosaic virus* (WSMV) is another persistent threat to wheat production. Transgenic wheat plants constitutively expressing a polycistronic cassette of five miR395 arms, known as FanGuard (FGmiR395), were exploited to target five distinct regions of the virus genome. The consequent transgenic plants showed nearly complete immunity to WSMV (Fahim et al. 2012). In the other case, a segment of 272 bp sequence derived from the coat protein of *Triticum mosaic virus* (TriMV) was cloned into the hairpin expression vector and constitutively expressed in wheat. The engineered wheat plants showed stable resistance to TriMV (Fahim et al. 2010).

### Management of wheat pests

Several major pests, such as grain aphid (*Sitobion avenae*), bird cherry-oat aphid (*Rhopalosiphum padi*), and wheat aphid (*Schizaphis graminum*), can cause severe yield loss (Table 1) (Peairs 2008; Smith and Chuang 2014; Yu et al. 2014). Transgenic wheat plants expressing a 198 bp fragment of dsRNA complementary to the zinc finger protein (*SaZFP*) of grain aphid can effectively increase its mortality and reduce its daily fecundity (Sun et al. 2019). In barley, dsRNA targeting the grain aphid gene encoding salivary sheath protein (SHP), a pivotal component of the stylet penetration process, effectively reduces the reproduction and survival rates of the aphid and the effect can be transmitted for seven generations (Abdellatef et al. 2015). Effects of additional target genes were also confirmed by feeding or direct injection into grain aphid, such as those encoding catalase, acetylcholinesterase1, cytochrome c oxidase subunit VIIc precursor, and zinc finger protein, and abnormally high mortality and developmental stunting were observed (Wang et al. 2015; Zhang et al. 2013).

### Management of nematodes in wheat

Wheat parasites cause enormous yield losses and threaten the quality of grains, including *Heterodera avenae*, *H. filipjevi* and *H. latipons* (Table 1) (Toumi and Waeyenberge 2013). Targeting of the *Ha18764* effector protein family genes of *H. avenae* by the VIGS-based RNAi approach significantly attenuated the parasitism and reproduction status of *H. avenae* in wheat (Yang et al. 2019). Down-regulation by RNAi of *pat-10* and *unc-87* genes on Thorne's meadow nematode (*Pratylenchus thornei*), which infects wheat roots, significantly reduced the reproduction of the worms (Tan et al. 2013)*.* Moreover, RNAi in wheat can be stimulated by poly-component biostimulants derived from metabolites of various soil streptomycetes which up-regulate siRNAs and miRNAs in wheat plants. These small RNAs are complementary to cereal cyst nematode mRNA and hence suppress their reproduction providing resistance to wheat plants (Blyuss et al. 2019).

## Challenges for using RNAi technology

While the outlook of using RNAi for plant protection appears to be promising, several issues need to be resolved before efficient practical applications.

### The stability of dsRNA

One of the primary concerns for the use of RNA as a biopesticide is their stability, especially for the sprayable dsRNA and siRNA applications. Microorganisms in the environment can degrade dsRNA prior to their uptake by pathogens or pests. Rapid degradation of dsRNA may occur by nucleases in the saliva, gut lumen, and/or haemolymph of pests as well (Allen and Walker III 2012; Chung et al. 2018; CoGuan et al. 2018; Katoch and Thakur 2012; Kennedy et al. 2004; Luo et al. 2013). The high or low pH found in the gut lumens of some pests can also reduce dsRNA stability either directly or indirectly by affecting the activity of gut nucleases (Cooper et al. 2019).

Other environmental factors may exert different effects on the stability of dsRNA and sRNA. Several works show that dsRNA is degraded to undetectable levels within 48 h after their application on three types of soil (silt loam, loamy sand, and clay loam) and within 7 days after their addition to aquatic systems containing natural water and various types of sediment (Albright et al. 2017; Fischer et al. 2017). Despite this, actin-dsRNA derived Colorado potato beetle (CPB) remained active for at least four weeks after application to potato leaves. It suppressed CPB larval weight gain, delayed its development, and increased its mortality (San Miguel and Scott 2016). Therefore, dissecting the process of dsRNA degradation is helpful in evaluating the potential effect of dsRNA in various environments and target organisms.

### Cost-effective methods for dsRNA production

For RNAi application to be practical for field use, the major hurdle is to produce sufficient amount of dsRNA. The traditional dsRNA production method in the laboratory is expensive and produces only a limited amount of dsRNA and thus is not practical for large-scale application needs (Ahn et al. 2019). Producing dsRNA in bacterial cells with RNaseIII deficiency seems to be an alternative. However, only a handful works have demonstrated microbial-based dsRNA production. One approach uses L1440-HT115 (DE3) system that has been successfully applied in the RNAi of *Mythimna separate* (Das et al. 2015; Parsons et al. 2018; Zhang et al. 2010). With more research underway, the production efficiency of this system should be augmented to meet market demands.

### Off-target effects

RNAi is a sequence homology-dependent mechanism. Several studies show that siRNA is not always specific and can have off-target effects and thus is problematic in disease management (Mamta and Rajam 2017). Some target genes are highly conserved between species which increases the likelihood of off-targets among them. The sequences of *vATPaseA* and *vATPaseE*from *L. decemlineata*, for instance, shared 83% and 79% nucleotide-sequence identities to their counterparts in Western Corn Rootworm (WCR), respectively. dsRNAs from WCR *vATPaseA* and *vATPaseE* could reduce the fitness of Colorado potato beetle (CPB; *Leptinotarsa decemlineata*) in a bioassay (Baum et al. 2007). Computational design program is needed for specific and systemic evaluation of non-target and off-target effects which should be further verified by additional bioassays. In addition, feeding studies revealed that dsRNAs of at least 60 nucleotide (nt) in length are necessary for an efficient RNAi response in *D. virgifera* (Bolognesi et al. 2012) and *Tribolium castaneum* (Wang et al. 2019). A minimum of 21 nt was required for the size of siRNA for efficient protection against WCR and active orthologs (Bachman et al. 2013).

### RNAi resistance

Pests and pathogens can develop resistance to RNAi-based products through various mechanisms as they do for conventional biopesticides. Compared to conventional commercialized transgenic crops expressing *Bt* toxins for pest management (James, 2010). RNAi-based strategy induces down-regulation of the target gene by in-complete resistance in most of cases. This may reduce the selection pressure on the pathogen that may contribute to durable resistance. But genetic variation in pathogenic organisms may also cause single nucleotide polymorphisms (SNPs) in the target gene. The efficiency of RNAi would be cut down owing to the reduction of complementarity between the target gene and the dsRNA. Synonymous SNPs lead to nearly no fitness cost on the pathogens and pests, but the difference between dsRNA and the original gene sequences reduces their complementarity, causing reduced RNAi effect or RNAi resistance (Scott et al. 2013; Yu et al. 2016). Thereby, the potential of RNAi resistance should be taken into consideration in application.

## Conclusions and future prospects

In the past few years, we have seen diverse applications of RNAi in crop protection methodologies against pests, pathogens, and nematodes. RNAi technology has emerged as a promising new strategy for wheat protection either. The wide use of HIGS on a commercial scale appears possible soon. The major obstacles for the HIGS strategy will be resolved, by optimal target and fragment selection methods, highly efficient transformation constructs, and stable transgenic systems. To this end, it is worthy to mention that the V-type ATPase-based RNAi technology has passed the GM safety evaluation in eight countries and regions including the United States, Brazil, and Japan. It has also been licensed for planting by the US Environmental Protection Agency (Zotti et al. 2018), painting an elusive picture for the commercialization of the RNAi technology. Technical barriers are being overcome to allow a wide range of applications from laboratory to the field. The technology of encapsulated dsRNA on leaves with SIGS has significantly promoted dsRNA stability in the environment as well as during its uptake by pests enhancing plant protection. Cost-effective approaches for massive production of dsRNA (e.g. bacterial, plant, and synthetic production) are being optimized and will contribute to lowering costs of the technology. There is no doubt that a new era of disease control based on RNAi technology for crop protection is right at the corner.
